# A Multiplex TaqMan-MGB qPCR Assay for Rapid and Accurate Identification of Four Waterfowl Parvoviruses (cGPV, MDPV, MDGPV, and SBDSV)

**DOI:** 10.1155/tbed/9999490

**Published:** 2025-09-28

**Authors:** Min Zheng, Dandan Jiang, Shifeng Xiao, Shao Wang, Xiaoxia Cheng, Xiaoli Zhu, Xiuqin Chen, Meiqing Huang, Shaoying Chen, Shilong Chen

**Affiliations:** ^1^Animal Virology Laboratory, Institute of Animal Husbandry and Veterinary Medicine, Fujian Academy of Agricultural Sciences, Fuzhou, China; ^2^Fujian Animal Diseases Control Technology Development Center, Fujian Academy of Agriculture Sciences, Fuzhou, China

**Keywords:** classical goose parvovirus, multiplex real-time pCR, Muscovy duck-origin goose parvovirus, Muscovy duck parvovirus, short beak and dwarfism syndrome virus, TaqMan-MGB probe

## Abstract

Waterfowl parvoviruses (WPVs), including classical goose parvovirus (cGPV), Muscovy duck parvovirus (MDPV), Muscovy duck-origin goose parvovirus (MDGPV), and short beak and dwarfism syndrome virus (SBDSV), are significant pathogens that affect waterfowl flocks worldwide. Due to their high genetic similarity and frequent coinfections, rapid and accurate differentiation of these viruses remains challenging. In this study, we developed a multiplex TaqMan-minor groove binder (MGB) real-time PCR assay for the simultaneous detection and differentiation of cGPV, MDPV, MDGPV, and SBDSV. Specific primers and TaqMan-MGB probes were designed based on sequence alignments of the VP gene. This assay exhibited high specificity, with no cross-reactivity to other main waterfowl viruses. The detection limits of this assay were 10^2^ copies/μL for cGPV, 10^1^ copies/μL for MDPV, 10^2^ copies/μL for MDGPV, and 10³ copies/μL for SBDSV, respectively. The standard curves exhibited strong linearity (R^2^≥0.995) and high amplification efficiency (89%–108%), with intra- and interassay coefficients of variation below 2.0%, indicating high repeatability and stability. Clinical testing of 337 clinical samples suspected of WPV infection demonstrated that the developed assay outperformed conventional PCR, achieving higher overall detection rates (58% vs 54%) and enhanced identification of coinfections. Epidemiological analysis revealed MDGPV as the predominant circulating strain in Muscovy ducks, with 27 samples identified as coinfected with both MDGPV and MDPV, while SBDSV showed higher prevalence in mule ducks and Pekin ducks. Notably, MDGPV was detected for the first time in goslings. These findings provide clear evidence of ongoing host restriction and potential cross-species transmission of WPVs among duck flocks. In conclusion, the multiplex TaqMan-MGB quantitative PCR (qPCR) assay developed in this study provides a rapid, sensitive, and reliable tool for the simultaneous detection and differentiation of cGPV, MDPV, MDGPV, and SBDSV. Its application is expected to enhance disease surveillance, facilitate outbreak control, and contribute to more effective control of waterfowl parvoviral diseases.

## 1. Introduction

Parvoviruses are small, nonenveloped DNA viruses that infect a wide range of animal species. Traditionally, their nomenclature has been based on the original host species rather than phylogenetic relationships [1]. According to the International Committee on Taxonomy of Viruses (ICTV), waterfowl parvoviruses (WPVs) belong to the family *Parvoviridae*, subfamily *Parvovirinae*, and genus *Dependoparvovirus* [2]. WPVs include classical goose parvovirus (cGPV), Muscovy duck parvovirus (MDPV), Muscovy duck-origin goose parvovirus (MDGPV), and short beak and dwarfism syndrome virus (SBDSV; also known as novel goose parvovirus, NGPV), all of which are major pathogens in global waterfowl production systems [3–5].

cGPV is the etiological agent of Derzsy's disease, characterized by exudative enteritis and intestinal obstruction in goslings and Muscovy ducklings, with early posthatch mortality rates reaching 90%–100% [6]. MDPV only infects young Muscovy ducks, causing enteritis, muscle degeneration, and growth retardation, and is particularly lethal in neonatal ducklings [7]. MDGPV is a recombinant virus of cGPV and MDPV under natural conditions. MDGPV was first isolated in southern China in 1997 from Muscovy duck farms. Pathogenicity studies have shown that MDGPV causes growth retardation, intestinal embolism and hemorrhage, and atrophic spleen in Muscovy ducklings, with clinical signs similar to Derzsy's disease caused by cGPV, with mortality rates ranging from 40% to 65% [8, 9]. Phylogenetic and epidemiological analyses suggest that MDGPV has become a key recombinant strain involved in mixed infections and may play a significant role in the ongoing evolution of WPVs [3].

Since 2015, SBDSV has emerged as an important pathogen in China, causing growth retardation and skeletal dysplasia in mule ducks and Pekin ducks, resulting in enormous economic losses [10, 11]. Compared to classical cGPV, SBDSV exhibits broader host tropism and more complex clinical manifestations, which further complicates their diagnosis and disease control [12, 13].

Frequent recombination and mutation events are hallmarks of WPV evolution, largely due to their compact single-stranded DNA (ssDNA) genomes and a propensity for coinfection in the same host [14]. These genetic dynamics profoundly influence viral antigenicity, virulence, host adaptation, and cross-species transmission [13, 15].

Mixed infections are increasingly reported in clinical settings, where not only different strains of WPVs cocirculate but also concurrent infections with other avian pathogens occur, posing serious challenges to disease surveillance and differential diagnosis [16, 17]. For example, Yang et al. [18] detected GPV and DuCV positivity rates of 82.8% and 78.9%, respectively, with a coinfection rate of 70% in the feather sacs of Cherry Valley ducks affected by feather shedding syndrome. Similarly, Liu et al. [19] further confirmed that GPV–DuCV coinfection is common in cases of short beak and dwarfism syndrome (SBDS), underscoring the clinical importance of mixed infections in waterfowl populations. Wang et al. [17] demonstrated, through the application of a quadruplex quantitative PCR (qPCR) assay to 396 samples, that single and mixed infection rates of MDPV, GPV, DuCV, and DAdV-3 were consistent with epidemiological patterns, highlighting the ongoing burden of multipathogen infections in duck farms. These findings underscore that mixed infections are not occasional but rather common in waterfowl production systems, further complicating clinical diagnosis. Due to the high genomic similarity among WPVs, accurate identification requires full genome sequencing, which is time-consuming and resource-intensive [20, 21]. To date, however, no clinical diagnostic method is available that can simultaneously and accurately differentiate all four WPV types.

In recent years, molecular diagnostic approaches for waterfowl viral diseases have advanced considerably, including the development of a quadruplex qPCR for MDPV/GPV with DuCV and DAdV-3 [17], rapid GPV detection using RPA-PfAgo [22], and field-deployable RPA-CRISPR/Cas12a assays for GPV [23], as well as multiplex qPCR strategies involving MDPV or NGPV with other waterfowl pathogens [24]. Nevertheless, despite these advances, no clinical diagnostic method currently exists that can simultaneously and accurately differentiate all four WPV types (cGPV, MDPV, MDGPV/NGPV, and SBDSV) within a single reaction. Addressing this gap is essential for reliable epidemiological monitoring, rapid outbreak response, and effective disease control in waterfowl production systems. To address this need, multiplex diagnostic approaches, particularly those based on TaqMan qPCR with minor groove binder (MGB) probe chemistry, offer substantial advantages. MGB probes enable precise discrimination between closely related viral sequences, including single-nucleotide polymorphisms (SNPs), and provide high specificity and thermal stability, making them highly suitable for multiplex applications [25, 26].

Given the high genomic similarity and overlapping epidemiology of cGPV, MDPV, SBDSV, and MDGPV, there is an urgent need for a robust, sensitive, and specific multiplex detection method for their simultaneous identification. In this study, we report the development and validation of a novel quadruplex TaqMan-MGB qPCR assay capable of detecting and differentiating cGPV, MDPV, SBDSV, and MDGPV. This assay demonstrates a highly analytical performance and provides a valuable molecular diagnostic tool for the comprehensive monitoring and early detection of WPV in waterfowl flocks.

## 2. Materials and Methods

### 2.1. Viral Strains and Clinical Samples

The cGPV strain NP5 (GenBank accession No. PQ272760), MDPV strain P (GenBank Accession Number KU844281), MDGPV strain D (GenBank Accession Number OQ301813), and SBDSV strain M15 (GenBank Accession Number OR777281) were isolated and preserved in our laboratory. Additional viral strains used as negative controls included duck adenovirus serotype B2 (DAdV-B2), duck Tembusu virus (DTMUV), duck enteritis virus (DEV), novel duck reovirus (NDRV), duck paramyxovirus (DPMV), Muscovy duck reovirus (MDRV), and duck hepatitis A virus type 1 (DHAV-1).

A total of 337 clinical samples suspected of WPV infection, including oropharyngeal and cloacal swabs, as well as liver, spleen, kidney, and intestinal tissues, were collected between 2021 and 2025 by veterinarians from duck and goose farms in southern China. All samples were submitted with the informed consent of farm owners, obtained through attending veterinarians for diagnostic purposes. Subsequent use of the samples for research was conducted in accordance with institutional and ethical guidelines.

### 2.2. Nucleic Acid Extraction

Total viral DNA and RNA were extracted from the clinical specimens and viral isolates using the Virus DNA/RNA Extraction Kit 2.0 (Vazyme Biotech, Nanjing, China) according to the manufacturer's protocol. The extracted nucleic acids were immediately stored at −20°C until further use.

### 2.3. Sequence Comparison and Primer Design

Complete genomic sequences of WPVs were retrieved from GenBank, including classical cGPV strains (B, Y, DY16, Y, NP5, YZ99-6, and LH); MDPV strains (P, YY, FZ91, FJM2, FJV1, and FJM5); MDGPV strains (D, PT, ZW, 2022JS, SAAS-SHNH, and GD201911); and SBDSV strains (sdlco1, SC16, AA, SD, AH, and M15). Multiple sequence alignment was conducted using DNASTAR software (DNASTAR, Madison, WI, USA) to identify conserved and divergent regions among the 18 WPV strains.

Based on the alignment results, specific primers and TaqMan-MGB probes targeting highly conserved regions of the VP gene sequences were designed using Primer Premier 5.0 software (Premier Biosoft, Palo Alto, CA, USA). The designed primers and probes were evaluated for specificity using the BLAST tool (NCBI), ensuring no cross-reactivity with nontarget sequences. Primer dimer formation and potential secondary structures such as hairpins were assessed and minimized using the PrimerSelect module of the DNASTAR software.

Multiple primer-probe sets were initially designed, and optimal combinations were selected through experimental screening to achieve the best amplification performance. In the final design, TaqMan-MGB probes were labeled with different fluorescent reporters at the 5' end: the cGPV probe was labeled with Cy5, the MDPV probe with VIC, the MDGPV probe with Texas Red, and the SBDSV probe with FAM. All probes were modified at the 3' end with an MGB group to enhance binding specificity and increase the melting temperature. The final sequences of primers and probes are detailed in Table 1. All primers and probes were synthesized by Sangon Biotech (Shanghai, China).

### 2.4. Construction of Standard Plasmids

Specific primer pairs targeting the VP gene regions of cGPV, MDPV, MDGPV, and SBDSV were designed to amplify the fragments containing the TaqMan-qPCR target sequences (primer sequences are listed in Table 2). PCR amplification was performed in a 20 μL reaction volume containing 10 μL of 2x Taq Master Mix (Dye Plus) (Vazyme Biotech, Nanjing, China), 2 μL of DNA template, 1 μL of each primer (10 μM), and 6 μL of nuclease-free water (ddH_2_O). The thermal cycling conditions were as follows: an initial denaturation at 95°C for 3 min, followed by 35 cycles of denaturation at 95°C for 15 s, annealing at 58°C for 15 s, extension at 72°C for 30 s, and a final extension at 72°C for 5 min.

The amplified PCR products were purified and cloned into the pMD18-T vectors (Takara Bio, Shiga, Japan) to generate the recombinant plasmids p-cGPV, p-MDPV, p-MDGPV, and p-SBDSV, respectively. The identity of the recombinant plasmids was confirmed by Sanger sequencing. The concentrations and purity of the plasmids were quantified using a DS-11 spectrophotometer (DeNovix, Wilmington, DE, USA). The copy number of each recombinant plasmid was calculated using the following formula:

Copies/*µ*L=(6.02 × 10^23^) × (X ng/*µ*L × 10^⁻9^)/(plasmid length(bp) × 660), 

where *X* represents the plasmid concentration in ng/μL. The plasmids were stored at −20°C until use.

### 2.5. Preparation of Mixed Plasmid Standards

Based on the calculated copy numbers, the individual positive control plasmids (1 × 10^10^ copies/μL) for cGPV, MDPV, MDGPV, and SBDSV were mixed in equal volumes with 1x TE buffer to prepare a mixed standard solution at a final concentration of 10^9^ copies/μL. Serial 10-fold dilutions were performed to generate a series of standards ranging from 10^0^ to 10^9^ copies/μL for assay sensitivity evaluation.

### 2.6. Conventional PCR

For the conventional PCR detection of the four WPVs (cGPV, MDPV, MDGPV, and SBDSV), the specific primers listed in Table 2 were used. The final reaction system was optimized in a total volume of 20 μL, containing 10 μL of 2x Taq Master Mix (Vazyme Biotech, Nanjing, China), 0.5 μL each of forward and reverse primers, 2 μL of mixed template DNA, and nuclease-free water to the final volume. The PCR amplification was performed under the following cycling conditions: an initial denaturation at 95°C for 30 s, followed by 35 cycles of denaturation at 95°C for 10 s, annealing at 53°C, and extension at 72°C for 1 min, with a final extension at 72°C for 5 min.

### 2.7. Establishment and Optimization of the Multiplex Real-Time PCR Reaction System

Based on the established singleplex real-time PCR conditions for each target, a multiplex real-time PCR assay was developed and optimized. The primer and probe concentrations were optimized using a matrix approach.

Optimization parameters included reaction temperature, primer concentration, and probe concentration. Primers and probes for cGPV, MDPV, MDGPV, and SBDSV were initially diluted to final concentrations ranging from 0.1 to 0.6 μM. Gradient PCR was performed with annealing temperatures ranging from 58 to 65°C to determine the optimal conditions.

Real-time PCR reactions were carried out under various combinations of primer/probe concentrations and annealing temperatures. The optimal reaction system was determined based on the amplification efficiency, specificity, and fluorescence signal strength, ultimately identifying the best primer and probe concentrations and the optimal annealing temperature for the multiplex assay.

### 2.8. Standard Curve Creation

The recombinant plasmids p-cGPV, p-MDPV, p-MDGPV, and p-SBDSV were serially 10-fold diluted to generate standard samples with concentrations ranging from 10^8^ to 10^3^ copies/μL. For each dilution point, three technical replicates were performed using the established multiplex real-time PCR assay. Standard curves were generated by plotting the logarithm of the initial plasmid copy number (*x*-axis) against the corresponding cycle threshold (Ct) values (*y*-axis). Regression analysis was conducted to establish quantitative standard curves for cGPV, MDPV, MDGPV, and SBDSV, respectively, providing the basis for assay sensitivity evaluation and absolute quantification.

### 2.9. Sensitivity, Specificity, and Repeatability Evaluation

#### 2.9.1. Specificity Evaluation

To assess assay specificity, nucleic acids extracted from the control strains, including DAdV-B2, DTMUV, DEV, NDRV, DPMV, MDRV, and DHAV-1, were used as templates. Normal Muscovy duck fibroblasts were included as a negative control. Specificity was evaluated by performing the multiplex TaqMan-MGB qPCR assay with these samples.

#### 2.9.2. Sensitivity Evaluation

To determine the sensitivity of the assay, recombinant plasmids p-cGPV, p-MDPV, p-MDGPV, and p-SBDSV were serially diluted 10-fold and subjected to multiplex qPCR until the templates could no longer be detected. The lowest detectable copy number for each target was recorded as the assay's limit of detection (LOD). In addition, the detection performance was compared with conventional PCR methods to evaluate improvements in sensitivity. The correlation between the dilution concentrations and qPCR positivity rates was also analyzed.

#### 2.9.3. Repeatability Evaluation

The intra- and interassay repeatability of the developed multiplex TaqMan-MGB qPCR assay was assessed by testing the plasmid standards (p-cGPV, p-MDPV, p-MDGPV, and p-SBDSV) at different time points in three independent experiments. For each assay, the Ct values of each target were recorded. The coefficient of variation (CV) was calculated using the formula: CV (%) = (standard deviation [SD]/mean [*X]*) × 100 to evaluate the assay's reproducibility and stability.

### 2.10. Detection of Clinical Samples

All clinical samples, including oropharyngeal swabs, cloacal swabs, and tissue specimens (liver, spleen, kidney, and intestine), collected from the same duck or geese were tested individually using both the multiplex TaqMan-MGB qPCR assay and conventional PCR. A waterfowl was considered positive for a specific pathogen if *any one* of its collected samples tested positive, in order to maximize detection sensitivity and to account for potential variations in viral distribution across different sample types.

## 3. Results

### 3.1. Validation of Primers and Probes

**Figure 1:**
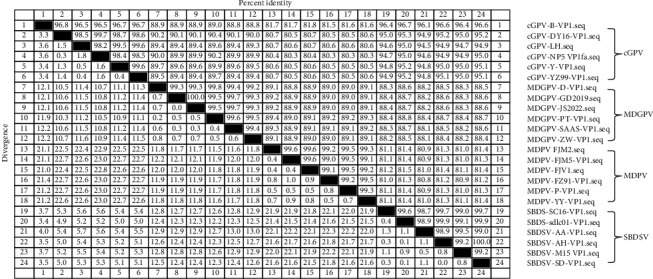
Comparative VP1 sequence homology of four waterfowl parvoviruses (cGPV, MDGPV, MDPV, and SBDSV).

**Figure 2:**
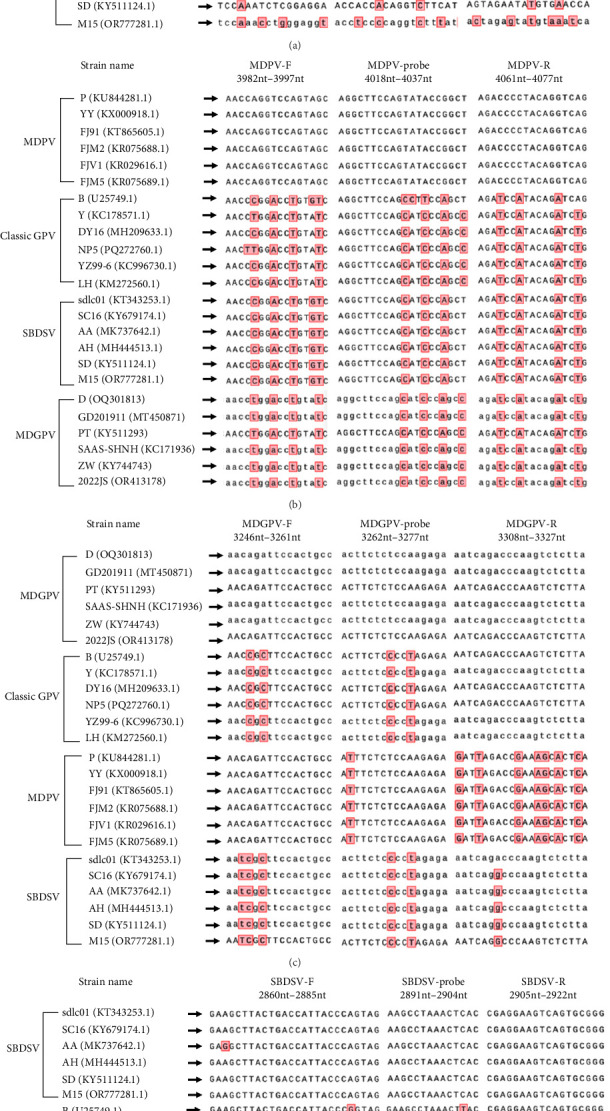
Nucleotide alignment of primer sequences from different members of waterfowl parvoviruses. The bases that differ from primer sequences are highlighted by background color. (A) Alignment of cGPV Primers and probe with MDPV, MDGPV, and SBDSV. (B) Alignment of MDPV Primers and probe with cGPV, MDGPV, and SBDSV. (C) Alignment of MDGPV Primers and probe with cGPV, MDPV, and SBDSV. (D) Alignment of SBDSV Primers and probe with cGPV, MDPV, and MDGPV.

Comparative analysis of VP1 sequences (Figure 1) showed that SBDSV shared the highest homology with cGPV (≈94%–97%), MDPV was the most divergent (≈80%–82%), and MDGPV displayed intermediate identity (~88%–90% with cGPV/SBDSV and ~89% with MDPV), consistent with its recombinant/intermediate characteristics. Based on this genomic framework, the designed primers and probes were aligned against the genomic sequences of other WPV strains to assess potential cross-reactivity with nontarget viruses. As shown in Figure 2, the cGPV TaqMan-qPCR primers (cGPV-F and cGPV-R) and probe (cGPV-P) exhibited high sequence conservation among cGPV strains (B, DY16, Y, NP5, YZ99−6, and LH). In contrast, multiple sequence variations were observed when compared with MDPV strains (P, YY, FZ91, FJM2, FJV1, and FJM5); MDGPV strains (D, PT, ZW, 2022JS, SAAS-SHNH, and GD201911); and SBDSV strains (sdlco1, SC16, AA, SD, AH, and M15). Similarly, the MDPV-, MDGPV-, and SBDSV-specific primers and probes each showed high sequence conservation within their respective viral strains but clear mismatches when aligned with other related parvoviruses (cGPV, MDGPV, MDPV, and SBDSV). These findings confirm that the designed primer-probe sets are highly specific to their respective target viruses and suitable for specific detection in the developed multiplex qPCR assay.

### 3.2. Establishment of the Multiplex Real-Time PCR Reaction System and Optimization of Reaction Conditions

Following systematic optimization, the final reaction system for the multiplex TaqMan-MGB real-time PCR assay was established in a total volume of 20 μL, comprising 10 μL of 2x All-Powerful qPCR PreMix (Vazyme Biotech, Nanjing, China), 0.4 μL each of the forward and reverse primers for cGPV, MDPV, MDGPV, and SBDSV (final concentration 0.2 μM per primer), 0.2 μL each of the corresponding TaqMan-MGB probes (final concentration 0.1 μM per probe), 2 μL of mixed template DNA, and nuclease-free water to complete the volume. The optimized thermal cycling conditions were as follows: contamination digestion 53°C for 10 min; initial denaturation 95°C for 30s; 45 cycles of denaturation at 95°C for 10 s and annealing/extension at 60°C for 20 s. These optimized conditions ensured efficient amplification and high specificity for the simultaneous detection of cGPV, MDPV, MDGPV, and SBDSV.

### 3.3. Standard Curve Preparation and Evaluation

**Figure 3:**
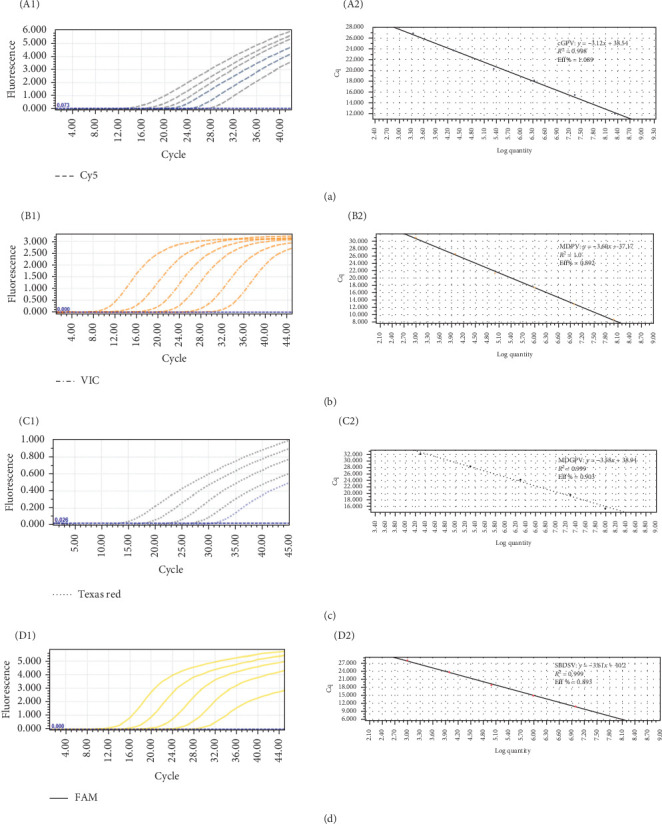
The amplification curves and standard curves of the TaqMan-MGB qPCR assay. The concentrations ranging from 10^8^ to 10^3^ copies/µL. (A; A1, A2), (B; B1, B2), (C; C1, C2), and (D; D1, D2) The amplification and standard curves of the standard plasmid of p-cGPV, p-MDPV, p-MDGPV, and p-SBDSV, respectively.

Standard plasmid solutions were serially diluted 10-fold with DNase/RNase-free water to concentrations ranging from 10^3^ to 10^8^ copies/μL. These dilutions were used as templates for the multiplex TaqMan-MGB real-time PCR assay. The logarithm of the initial plasmid copy number (*x*-axis) was plotted against the corresponding Ct values (*y*-axis) to generate standard curves for cGPV, MDPV, MDGPV, and SBDSV, respectively, as shown in Figure 3.

The linear regression equations and assay performance parameters indicated strong assay efficiency and consistency. For cGPV, the standard curve regression equation was *y =* –3.12*x* + 38.54, with an amplification efficiency of 108.9% and a correlation coefficient (*R*^2^) of 0.998. For MDPV, the equation was *y =* –3.60*x* + 37.17, with an amplification efficiency of 89.2% and an *R*^2^ of 1.000. The regression equation for MDGPV was *y =* –3.58*x* + 38.94, indicating 90.3% amplification efficiency and an *R*^2^ of 0.999. For SBDSV, the regression equation was *y =* –3.61*x* + 40.20, with an amplification efficiency of 89.3% and an *R*^2^ of 0.999. These results confirm that the established multiplex TaqMan-MGB qPCR assay provides strong correlation and high amplification efficiency across a broad dynamic range of template concentrations, enabling accurate quantification of viral loads in unknown samples by substituting their Ct values into the corresponding equations.

### 3.4. Specificity Evaluation

To evaluate the specificity of the developed multiplex TaqMan-MGB qPCR assay, DNA or cDNA extracted from eight major waterfowl viruses, including DAdV-B2, DTMUV, DEV, NDRV, DPMV, MDRV, and DHAV-1, was tested as templates. The nucleic acids of cGPV, MDPV, MDGPV, and SBDSV served as positive controls, while DNA extracted from normal Muscovy duck fibroblasts was used as a negative control.

**Figure 4:**
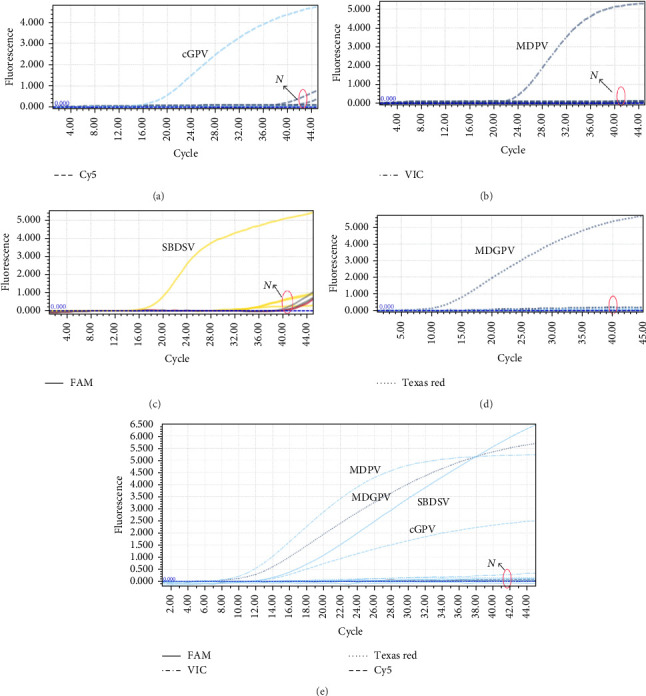
The specificity of the multiplex TaqMan-MGB qPCR assay. Amplification plots are shown for (A) cGPV (Cy5 channel), (B) MDPV (VIC channel), (C) SBDSV (FAM channel), (D) MDGPV (Texas Red channel), and (E) simultaneous detection of cGPV, MDPV, MDGPV, and SBDSV in a single reaction (Cy5, VIC, Texas Red, and FAM channels, respectively). The N denotes negative controls used in this study, including DAdV-B2, DTMUV, DEV, NDRV, DPMV, MDRV, DHAV-1, and ddH_2_O. No positive signals were detected in the controls.

The results showed that fluorescence signals were exclusively detected in the corresponding channels: Cy5 for cGPV, VIC for MDPV, Texas Red for MDGPV, and FAM for SBDSV. No amplification signals were observed from the other eight viruses and the negative control, indicating that the assay exhibited high specificity for the detection of the 4 WPVs (Figure 4A-D). Additionally, when the nucleic acids of cGPV, MDPV, SBDSV, and MDGPV were mixed and tested simultaneously, specific amplification signals were observed only in their respective channels without any cross-reactivity, further confirming the excellent specificity and reliability of the multiplex assay (Figure 3E).

### 3.5. Sensitivity Evaluation

**Figure 5:**
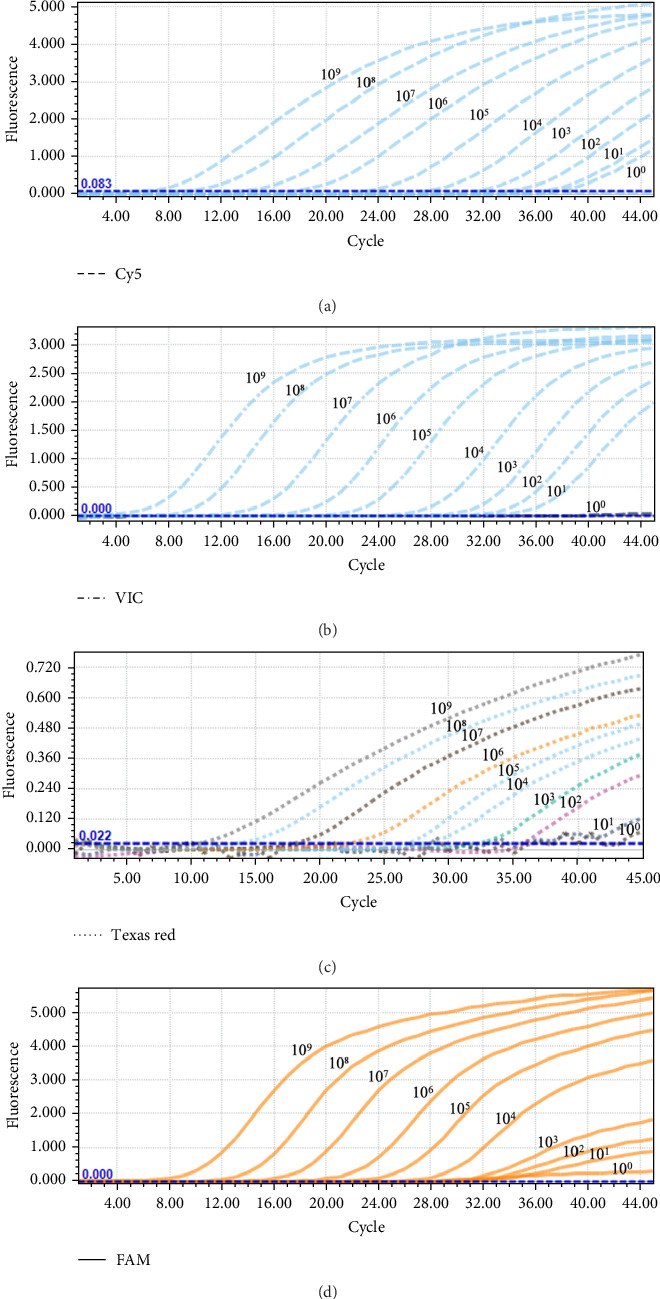
Amplification curves of 10-fold serial dilutions of standard plasmids for sensitivity evaluation of the multiplex TaqMan-MGB qPCR assay. The dilution range was 10^9^ to 10^0^ copies/μL. (A) cGPV detection using the Cy5 channel; (B) MDPV detection using the VIC channel; (C) MDGPV detection using the Texas Red channel; and (D) SBDSV detection using the FAM channel. The assay reliably detected cGPV, MDPV, MDGPV, and SBDSV at minimum concentrations of 10^2^, 10^1^, 10^2^, and 10^3^ copies/μL, respectively.

To evaluate the sensitivity of the developed multiplex TaqMan-MGB real-time PCR assay, standard plasmids p-cGPV, p-MDPV, p-MDGPV, and p-SBDSV were subjected to 10-fold serial dilutions ranging from 10^9^ to 10^0^ copies/μL and used as templates in the assay. The results demonstrated that the assay could reliably detect as low as 10^2^ copies/μL for cGPV, 10^1^ copies/μL for MDPV, 10^2^ copies/μL for MDGPV, and 10^3^ copies/μL for SBDSV (Figure 5), indicating high sensitivity across all 4 targets.

According to the established criteria for result interpretation, samples with a Ct value less than 38 and exhibiting a typical S-shaped amplification curve are considered positive. Samples with Ct values ≥38 or no Ct value and lacking a characteristic amplification curve are considered negative.

### 3.6. Repeatability Evaluation

To assess the repeatability of the multiplex TaqMan-MGB real-time PCR assay, plasmid standards of p-cGPV, p-MDPV, p-MDGPV, and p-SBDSV at three different dilution gradients (10^7^ to 10^5^ copies/μL) were selected to test intra-assay and interassay variation. The CV for the Ct values of each replicate was calculated to assess reproducibility. As shown in Table 3, the intra-assay CV for p-cGPV, p-MDPV, p-MDGPV, and p-SBDSV ranged from 0.53% to 1.90%. Similarly, the interassay CVs ranged from 0.66% to 1.69%. All intra- and interassay CV values were below 2%, indicating that the developed multiplex qPCR assay exhibits excellent repeatability and stability across different template concentrations.

### 3.7. Detection of Clinical Samples

**Figure 6:**
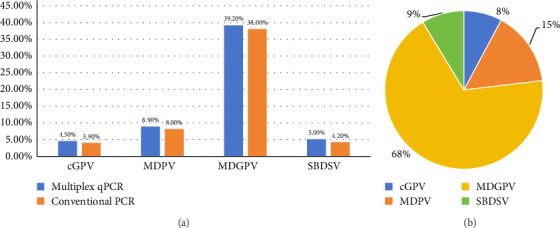
(A) Comparison of detection rates of cGPV, MDPV, MDGPV, and SBDSV in clinical samples using multiplex qPCR versus conventional PCR. (B) Genotypic distribution of waterfowl parvoviruses among qPCR-positive samples (*n* = 194) detected using the multiplex TaqMan-MGB qPCR assay.

The established multiplex TaqMan-MGB qPCR assay was systematically applied to 337 clinical samples collected from waterfowl farms in southern China between 2021 and 2025, including 269 samples from Muscovy ducks, 18 from mule ducks, 22 from Pekin ducks, and 28 from geese. Parallel testing with conventional PCR demonstrated superior performance of the multiplex qPCR assay, with an overall positivity rate of 58% (194/337) compared to 54% (182/337) for conventional PCR. Virus typing results demonstrated higher detection rates by multiplex qPCR compared to conventional PCR: cGPV (4.5% [15/337] vs. 3.9% [13/337], exclusively in goose samples), MDPV (8.9% [30/337] vs. 8.0% [27/337]), MDGPV (39.2% [132/337] vs. 38% [128/337]), and SBDSV (5.0% [17/337] vs. 4.2% [14/337]), as shown in Figure 6A. Among all WPV–positive samples, MDGPV was identified as the predominant circulating strain in Muscovy ducks, accounting for 68% (132/194) of positive cases, followed by MDPV at 15% (30/194) (Figure 6B). Notably, three MDGPV-positive cases were detected in geese for the first time, suggesting that this genotype may be evolving enhanced adaptability to a broader range of waterfowl hosts. In addition to improving the detection of single-genotype infections, the multiplex assay also enabled accurate identification of mixed infections, with 27 samples found to be coinfected with both MDGPV and MDPV. To further validate these results, sequencing was performed on qPCR-positive but cPCR-negative samples, including cGPV (2 samples), MDPV (3 samples), MDGPV (4 samples), SBDSV (3 samples), as well as the 27 coinfected samples. The amplified PCR products were purified and cloned into pMD18-T vectors (Takara Bio, Shiga, Japan) for sequencing. The sequencing results confirmed the presence of the corresponding viral sequences in all tested samples, demonstrating that the higher positivity rate observed with qPCR reflects its superior sensitivity rather than false-positive amplification. Overall, these findings underscore the value of this multiplex TaqMan-MGB qPCR method for comprehensive surveillance of WPVs. The improved sensitivity, specificity, and ability to differentiate among genotypes and detect coinfections provide a robust foundation for future epidemiological monitoring and targeted disease control strategies.

## 4. Discussion

**Figure 7:**
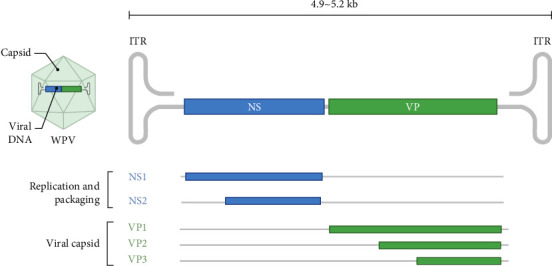
Schematic diagram of the genomic organization of waterfowl parvoviruses (WPVs).

WPVs possess a unique genomic structure characterized by inverted terminal repeat (ITR) sequences at both ends, forming hairpin structures essential for viral replication. Their genomes are linear ssDNA, ~5.0–5.3 kb in length, and contain two major open reading frames (ORFs) [27, 28]. The left ORF encodes two nonstructural proteins, NS1 (Rep1) and NS2 (Rep2), which are critical for viral replication, transcriptional regulation, and particle assembly, and exhibit cytotoxic effects in host cells [14]. The right ORF encodes structural proteins VP1, VP2, and VP3, which are central to immunogenicity, tissue tropism, pathogenicity, and host specificity [14, 29] (Figure 7).

Classical GPV and MDPV share an overall nucleotide identity of 80.6%–82.6%, with 80.7%–83.0% homology in NS proteins and 79.6%–81.6% in VP proteins [28]. Because of their high morphological, physicochemical, and high genetic similarities, accurate differentiation between cGPV and MDPV is challenging [30]. SBDSV, also known as NGPV, is a cGPV variant lineage. It shares 91.5%–98.7% genomic identity with cGPV strains, with 93.4%–96.9% homology in NS proteins and 90.9%–96.7% in VP proteins [12]. These differences are demonstrated as scattered point mutations. While SBDSV is genetically close to cGPV, it forms a distinct phylogenetic clade, indicating a separate evolutionary path and unique host adaptation strategy [31].

MDGPV is a recombinant virus between MDPV and cGPV. MDGPV took the MDPV genome as the backbone, and the regions of the P9 promoter to NS (425–612 nt), NS2 (1,483–1,824 nt), and VP3 (3,124–4,248 nt) were replaced by the corresponding regions of cGPV, respectively or simultaneously [32]. MDGPV strains have a high pathogenicity in Muscovy ducks and cause substantial economic losses to the Chinese duck industry [33].

Under the selective pressures of host immunity and environmental factors, WPVs continue to evolve, giving rise to variants with expanded host ranges and altered pathogenicity [34]. A marked increase in coinfections and cross-species transmission events in WPV has caused the difficulty in clinical diagnosis and disease control [19, 35]. Consequently, accurate early diagnosis and continuous monitoring of infection are vital for understanding WPV evolution and guiding control measures.

To date, singleplex PCR or qPCR assays targeting individual WPVs have been developed [36, 37]. In addition, existing multiplex qPCR approaches remain largely limited to either differentiating two WPV species or detecting a single WPV species in combination with other pathogens [38, 39]. However, due to the high genetic similarity among WPVs, no method has yet been reported that enables the simultaneous and precise differentiation of all four WPV species within a single assay. These diagnostic gaps highlight the urgent need for advanced molecular tools to improve pathogen surveillance and disease control in waterfowl populations. In this study, we developed a novel multiplex TaqMan-MGB qPCR assay using highly specific primers and MGB-modified probes targeting the VP1 gene of cGPV, MDPV, MDGPV, and SBDSV. Given the high sequence similarity between SBDSV and cGPV (>90%) [20], designing specific primers to differentiate these two viruses was particularly challenging. However, the use of MGB probes, capable of distinguishing SNPs, effectively resolved this issue. Notably, the SBDSV- and cGPV-specific probes differ by only one nucleotide. The MGB modification significantly improves probe thermal stability and mismatch discrimination, enabling high-precision, simultaneous detection of closely related viral species.

The developed multiplex assay in this study reliably distinguished cGPV, MDPV, MDGPV, and SBDSV without cross-reactivity, even in samples containing mixed viral nucleic acids. However, conventional PCR methods often fail to distinguish between highly homologous viral strains. Additionally, the use of TaqMan-MGB chemistry enhances both specificity and sensitivity by increasing probe melting temperature (*T*_m_) and enabling detection of minor sequence differences. The detection limits of the assay ranged from 10^1^ to 10^3^ copies/μL, surpassing the sensitivity of conventional gel-based PCR methods. Intra- and interassay variability was minimal (CV < 2%), ensuring consistent detection even at low viral loads, a crucial feature for early diagnosis and subclinical monitoring.

Application of the assay to 337 clinical samples collected over 5 years from waterfowl farms in southern China revealed a higher overall detection rate than conventional PCR (58% vs. 54%). Epidemiological analysis further demonstrated that MDGPV, a novel recombinant virus derived from MDPV and cGPV, has emerged as the dominant circulating strain, with a detection rate of 68% (132/194). The recombinant nature of MDGPV may be closely linked to its increasing ecological adaptability and expanding host range. Notably, MDPV retained strict host specificity and was detected exclusively in Muscovy ducks. However, only three samples showed MDPV single infections, while most MDPV-positive samples (27/30) were coinfected with MDGPV. This pattern suggests a potential synergistic infection mechanism between the two viruses, possibly driven by convergent host tropism or underlying recombination events. These findings highlight the complex interplay between viral genetics and host specificity in shaping infection dynamics.

In contrast, cGPV exhibited strict host restriction, being detected only in goose-derived samples. Although its overall prevalence was lower, its continued circulation remains a concern for goose farming. Meanwhile, SBDSV was detected in 5.0% (17/337) of samples, primarily from mule ducks and Pekin ducks, suggesting ongoing transmission among Pekin-type meat ducks and a potential risk for future outbreaks. Collectively, these clinical findings underscore the persistent threat posed by WPVs to the duck and goose industries. The diversity of circulating genotypes, coupled with differences in host specificity and clinical manifestation, presents significant challenges for disease control. The multiplex qPCR assay developed in this study provides a rapid and reliable tool for genotype-specific detection and differentiation, enabling timely surveillance and supporting the implementation of targeted prevention strategies against the evolving landscape of waterfowl parvoviral infections.

## 5. Conclusion

In this study, we successfully developed and validated a multiplex TaqMan-MGB real-time PCR assay for the simultaneous detection and differentiation of four clinically significant WPVs: cGPV, MDPV, MDGPV, and SBDSV. The assay demonstrated excellent specificity, high sensitivity, strong linearity, and reliable repeatability. Compared with conventional PCR, the multiplex assay exhibited significantly higher detection rates and superior capability in identifying mixed infections.

## Figures and Tables

**Table 1:** Primers and probes used in the qPCR assay for the simultaneous detection of cGPV, MDPV, MDGPV, and SBDSV.

Primes	Sequence (5′ → 3′)	Primer location (nt)	Target gene of the reference strain	Product size (bp)
cGPV-F	TCCGAATCTCGGAGGA	4292–4307	NP5 (PQ272760)VP1	66
cGPV-R	TGGTGCACGTATTCTACT	4373–4390		
cGPV-PROBE	Cy5- ACCACCGCAGGTGTTCAT-MGB	4325–4342		

MDPV-F	AACCAGGTCCAGTAGC	3982–3997	P (KU844281) VP1	96
MDPV-R	CTGACCTGTAGGGGTC	4061–4077		
MDPV-PROBE	VIC-AGGCTTCCAGTATACCGGCT-MGB	4018–4037		

MDGPV-F	AACAGATTCCACTGCC	3246–3261	D (OQ301813) VP1	82
MDGPV-R	TAAGAGACTTGGGTCTGATT	3308–3327		
MDGPV-PROBE	TXR -ACTTCTCTCCAAGAGA-MGB	3262–3277		
SBDSV-F	GAAGCTTACTGACCATTACCCAGTAG	2860–2885	M15 (OR777281) VP1	63
SBDSV-R	CCCGCACTGACTTCCTCG	2905–2922		
SBDSV-PROBE	FAM-AAGCCTAAACTCAC-MGB	2891–2904		

**Table 2:** Primers used for recombinant plasmid standards construction.

Pathogens	Target gene	Primers	Sequence (5′ → 3′)	Product size (bp)	Accession number
cGPV	VP3	F	ATGGTTTGGCAGAACA	223	PQ272760.1
		R	ACTGGCCCGTAGAGTA		

MDPV	VP3	F	AGGAATGCACAGTTCAA	351	KU844281.1
		R	ATAGCACCAACTAATGGT		

MDGPV	VP1	F R	GTGGAACCTCTCAAGA TGATCCTGCGTTGTGA	381	OQ301813.1

SBDSV	VP1	F	AGTAGAAGAGCCTATCA	240	OR777281.1
		R	ATGGAGTGGGTAATGCC		

**Table 3:** Intra-assay and interassay repeatability of the multiplex TaqMan-MGB qPCR assay for cGPV, MDPV, MDGPV, and SBDSV at three different plasmid concentrations. Each plasmid standard was tested in triplicate across three runs. The coefficient of variation (CV) was calculated from the mean cycle threshold (Ct) values and standard deviation (SD).

Plasmid standards	Concentration of template (copies/μL)	Intracoefficient of variation		Intercoefficient of variation	
		X ± SD	CV (%)	X ± SD	CV (%)
p-GPV	10^7^	12.643 ± 0.240	1.90	12.740 ± 0.095	0.75
	10^6^	16.573 ± 0.167	1.00	16.213 ± 0.143	0.89
	10^5^	21.787 ± 0.170	0.78	21.587 ± 0.162	0.75

p-MDPV	10^7^	12.170 ± 0.155	1.28	12.627 ± 0.159	1.26
	10^6^	17.433 ± 0.179	1.03	17.687 ± 0.117	0.66
	10^5^	21.350 ± 0.125	0.59	21.707 ± 0.260	1.20

p-MDGPV	10^7^	18.170 ± 0.151	0.83	18.477 ± 0.264	1.43
	10^6^	22.727 ± 0.172	0.76	22.557 ± 0.273	1.21
	10^5^	27.310 ± 0.322	1.18	27.507 ± 0.342	1.24

p-SBDSV	10^7^	14.713 ± 0.122	0.83	14.517 ± 0.246	1.69
	10^6^	19.156 ± 0.106	0.55	19.620 ± 0.160	0.82
	10^5^	23.767 ± 0.125	0.53	24.180 ± 0.229	0.95

## Data Availability

The datasets supporting the conclusions of this study are available from the corresponding author upon reasonable request.
